# *Parvimonas micra* infection enhances proliferation, wound healing, and inflammation of a colorectal cancer cell line

**DOI:** 10.1042/BSR20230609

**Published:** 2023-06-13

**Authors:** Muhammad Nur Adam Hatta, Ezanee Azlina Mohamad Hanif, Siok-Fong Chin, Teck Yew Low, Hui-min Neoh

**Affiliations:** UKM Medical Molecular Biology Institute (UMBI), Universiti Kebangsaan Malaysia, Jalan Ya’acob Latiff, Cheras, Kuala Lumpur 56000, Malaysia

**Keywords:** colorectal cancer, HT-29, infection, iTRAQ-LC-MS/MS, Parvimonas micra, pathogenesis

## Abstract

The gut microbiota *Parvimonas micra* has been found to be enriched in gut mucosal tissues and fecal samples of colorectal cancer (CRC) patients compared with non-CRC controls. In the present study, we investigated the tumorigenic potential of *P. micra* and its regulatory pathways in CRC using HT-29, a low-grade CRC intestinal epithelial cell. For every *P. micra*-HT-29 interaction assay, HT-29 was co-cultured anaerobically with *P. micra* at an MOI of 100:1 (bacteria: cells) for 2 h. We found that *P. micra* increased HT-29 cell proliferation by 38.45% (*P*=0.008), with the highest wound healing rate at 24 h post-infection (*P*=0.02). In addition, inflammatory marker expression (*IL-5*, *IL-8*, *CCL20*, and *CSF2*) was also significantly induced. Shotgun proteomics profiling analysis revealed that *P. micra* affects the protein expression of HT-29 (157 up-regulated and 214 down-regulated proteins). Up-regulation of PSMB4 protein and its neighbouring subunits revealed association of the ubiquitin–proteasome pathway (UPP) in CRC carcinogenesis; whereas down-regulation of CUL1, YWHAH, and MCM3 signified cell cycle dysregulation. Moreover, 22 clinically relevant epithelial–mesenchymal transition (EMT)-markers were expressed in HT-29 infected with *P. micra*. Overall, the present study elucidated exacerbated oncogenic properties of *P. micra* in HT-29 via aberrant cell proliferation, enhanced wound healing, inflammation, up-regulation of UPPs, and activation of EMT pathways.

## Background

Unhealthy diet and lifestyle, which have long been associated with the occurrence of CRC, can also lead to microbiota dysbiosis in the colon [1,2]. As a result of dysbiosis, pathogenic microbes displace normal flora in the colon and/or rectum, disrupting its normal regulation and homeostasis [3,4]. Specifically, settlement of these pathobionts may trigger the secretion of toxins, activation of reactive oxygen species (ROS), and inflammation of colon cells; thereby increasing the risks of CRC in the host [5]. Indeed, scientists have proposed the tumorigenic roles of gut-associated microbes in CRC, including *Streptococcus bovis* [6], *Enterococcus faecalis* [7], *Bacteroides fragilis* [8], *Streptococcus gallolyticus* [9], *Escherichia coli* [10], and *Fusobacterium nucleatum* [11].

Recently, we reported a 3-fold enrichment of *Parvimonas micra*, an oral bacteria, in the gut mucosa tissues of 18 Malaysian CRC patients compared with 18 non-CRC patients based on 16S rRNA amplicon sequencing [12]. Though the roles of *P. micra* in CRC oncogenesis remain unclear, our previous findings were consistent with published meta-analyses, which demonstrated enrichment of *P. micra* in almost 2,000 participants of various CRC cohorts from different geographical locations [13,14]. Besides CRC, *P. micra* infections have also been associated with cancer of the oral cavity and stomach [15,16], underscoring the intriguing association and possible role of the bacteria in malignant tumour occurrence and development.

Later, Zhao and co-workers’ studies investigating *P. micra* infection using *in vivo* models indicated positive correlations of *P. micra* abundance with tumour burden, cell proliferation, and the expression of pro-inflammatory cytokines [17,18]. In this work, we elected instead to follow up in an *in vitro* manner our previous findings by infecting HT-29, a CRC intestinal epithelial cell line with *P. micra*. We aimed to investigate the effects of this infection on cell morphology and functional characteristics. We additionally profiled the molecular changes of the proteome as a result of this bacteria–host cell interaction with shotgun proteomics followed by pathway analysis, to provide a better understanding of the role of *P. micra* in CRC.

## Hypothesis

We hypothesized that *P. micra* infections might play a role in CRC pathogenesis in the HT-29 cell line by affecting its cell proliferation, wound healing, cell cycle activity, and causing inflammation. From this study, additional information of the putative tumorigenic role of *P. micra* in CRC would be elucidated.

## Methodology

### Bacteria culture

*P. micra* (DSM 20468) was purchased from DSMZ-German Collection of Microorganisms and Cell Cultures GmbH (Germany) in active culture form. *E. coli* DH5α was a gift from the Department of Bacteriology, Juntendo University, Tokyo, Japan. Both bacteria were cultured at 37°C in Brain Heart Infusion (BHI) broth anaerobically in an AnaeroPack™ 2.5 L rectangular jar containing an AnaeroGen™ sachet 3.5 L [19] for all experiments.

### Intestinal epithelial cell culture

A low-grade colon cancer intestinal epithelial cell, HT-29 was used in all experimental setups. It was purchased from American Type Culture Collection (ATCC, U.S.A.) and maintained using the Roswell Park Memorial Institute (RPMI) 1640 media (Pan Biotech, Germany) supplemented with 12% fetal bovine serum (Tico Europe, Netherlands). For co-culture assays, 1% penicillin/streptomycin (Nacalai Tesque, Inc., Japan) was added post-infection to stop bacterial growth. For the maintenance of the cells, HT-29 was cultured inside a 37°C incubator supplemented with 5% CO_2_, and 95% O_2_. During co-culture assays, the cells were maintained in a similar anaerobic condition as the tested bacteria [20].

### HT-29 and bacteria co-culture

#### Cell proliferation assay

HT-29 cells were seeded in a 24-well plate at a density of 5 × 10^4^ cells per well in antibiotic-free growth media and grown aerobically overnight. After overnight incubation, cells were introduced with *P. micra* at a multiplicity of infection (MOI) of 100:1 (bacteria: cells) [21]. *E. coli* DH5α was used as bacteria-host interaction control, while uninfected HT-29 was a control for the co-culture system. The plate was incubated at 37°C for 2 hr anaerobically. Next, all experiment sets were washed three times with 1× PBS solution followed by supplementation of growth media with antibiotics. The plate was then incubated for another 72 hr anaerobically. Subsequently, cells were fixed with 20% methanol and stained with crystal violet stain solution (Merck, Germany). Cell images were taken with a ChemiDoc MP Imaging System (Bio-Rad, U.S.A.). Dried stained cells were then diluted with 10% acetic acid prior optical density (OD) reading at 600 nm using a Varioskan™ Flash Spectral Scanning Multimode Reader (Thermo Fisher Scientific, U.S.A.). All experiments were conducted three times with technical triplicates.

#### Wound healing assay

Twelve-well plates were marked on the bottom of each well at specific locations prior experiments. HT-29 cells were then seeded at a total of 5 × 10^5^ cells/well. Cells were supplemented with antibiotic-free growth media and grown overnight. Wells with at least 90% confluent cells were later wounded using 200 μl pipette tips and washed with 1× PBS solution. Wounded cells were then replenished with antibiotic-free growth media. Tested bacteria were introduced to the cells at an MOI of 100:1 (bacteria: cells); uninfected cells were introduced to BHI broth only. Co-cultured plates were incubated at 37°C for 2 hr anaerobically. Later, cells were washed three times with 1× PBS solution and replenished with growth media supplemented with antibiotics. Wound images were observed and captured at three different time points (0, 24, and 48 hr) at a unified location. Wound area size was measured as the distance between cell edges using ImageJ Software Version 1.53f (Maryland, U.S.A.) with the value at 0 hr as a baseline. The difference in wound size percentage between each tested bacteria–host interaction assay and the control was also determined. Each experiment setup was repeated three times with technical triplicates.

#### Inflammatory marker expression

HT-29 cells were seeded in a P60 cell culture dish at a density of 5 × 10^5^ cells per dish. Cells were then grown overnight anaerobically. Adhered cells were infected with *P. micra* at an MOI of 100:1 (bacteria: cells). Uninfected cells supplemented with BHI broth were used as a control. Both infected and uninfected HT-29 were incubated anaerobically at 37°C for 2 hr. Later, the growth media was moved and cell monolayers were washed three times with 1× PBS. Cells were then replenished with growth media supplemented with antibiotics, and further cultured anaerobically for 2, 24, or 48 hr. At the end of each designated incubation period, total cell RNA from each experiment set was extracted using guanidinium thiocyanate–phenol–chloroform extraction. Extracted RNA was converted to cDNA using a High-Capacity cDNA Reverse Transcription Kit (Applied Biosystems, U.S.A.). Relative expression of inflammatory genes (*IL-5*, *IL-8*, *IL-22*, *CCL20*, *TIMP1*, *CSF2*) and housekeeping genes (*HPRT*, *β*-*tubulin*) was analysed using QuantiNova SYBR® Green PCR Kit (QIAGEN, U.S.A.) via an Applied Biosystem® 7500 Fast Real-Time PCR (Thermo Fisher Scientific, U.S.A.). Primer sequences for each tested gene were designed using the Primer-BLAST software (http://www.ncbi.nlm.nih.gov/tools/primer-blast) (NCBI, U.S.A.) and listed in Table 3.

#### Differential proteomics profiling

Proteomics analysis of HT-29 infected with *P. micra* was determined to elucidate pathways associated with *P. micra*-associated CRC development. HT-29 cells were seeded in a 6-well plate at a density of 5×10^5^ cells/mL and incubated aerobically overnight. Cells were then co-cultured with *P. micra* at an MOI of 100:1 (bacteria: cells), whereas uninfected cells were supplemented with BHI. Both setups were incubated anaerobically for 2 hr. After 2 hr, cells were washed with 1× PBS three times and replenished with growth media supplemented with antibiotics. Cells were incubated again anaerobically for 24 hr and harvested by adding 2× of 0.25% Trypsin-EDTA solution followed by centrifugation at 200 *× **g*** for 5 min.

Cell pellets were subsequently collected for protein extraction and proteomics analysis. Total cell protein was extracted using lysis buffer (8 M urea, 2 M thiourea, 50 mM ammonium bicarbonate) with pulsed ultra-sonication and high-speed centrifugation. Protein concentration was estimated using a Bradford assay. Extracted proteins were later subjected to acetone precipitation. Protein reduction, alkylation, trypsin digestion, iTRAQ labelling (AB SCIEX, U.S.A.), C18 reverse-phase desalting, and LC-MS/MS analysis with the Eksigent reversed-phase nanoLC (AB SCIEX, U.S.A.) coupled with 6600 TripleTOF (AB SCIEX, U.S.A.) system were carried out by the Protein and Proteomic Center (PPC), Department of Biological Sciences, Faculty of Science, National University of Singapore (NUS) using the standard protocol in their previous study [22].

For analysis, protein sequences were extracted from ProteinPilot 5.0 software Revision 4769 (AB SCIEX, U.S.A.), by using the Paragon database search algorithm (5.0.0.0.4767) and integrated false discovery rate (FDR) analysis function. Later, protein profiles were compared with the *Homo sapiens* reference proteome (UP000005640, 29 Jan 2021, 20380 entries) using SwissProt and spiked contaminant proteins (cRAP). A global FDR threshold at 1% was implemented for differential protein determination (at least one peptide). Protein log2 fold change with a value of >1.0 was categorized as up-regulated, whereas a log2 fold change of < -1.0 was considered as down-regulated. Lastly, all proteins without iTRAQ quantitation ratios, and/or with accession and with ‘contam’, ‘RRRR’ and ‘REVERSED’ descriptions were removed from the analysis.

### *P. micra*-associated CRC tumorigenesis pathway determination

DAVID Bioinformatics Resources 6.8 database (https://david.ncifcrf.gov/) and PANTHER16.0 database (http://pantherdb.org/) were used to determine Gene Ontology (GO) classification of identified proteins based on biological processes, molecular functions, and cellular components. Pathway analyses were performed using the Kyoto Encyclopaedia of Genes and Genomes (KEGG) database. Protein interactions were built using STRING 11.5 databases (https://string-db.org/) for selected proteins. Identification of EMT-related proteins was done using EMTome (http://www.emtome.org/) database and dbEMT 2.0 database (http://dbemt.bioinfo-minzhao.org/).

### Statistical analysis

All data obtained were analysed using GraphPad PRISM 9.2.0 (GraphPad Software, U.S.A.). The Mann–Whitney test, two-way multiple comparisons ANOVA and Student’s *t*-test were used to analyze results from functional assays, inflammatory marker expression determination and differential proteomics profiling, respectively. All statistical analyses with *P*-values of *≤ 0.05, **≤ 0.01, ***≤0.001, and ****≤ 0.0001 were considered significant.

## Results

### *P. micra* increased the rate of HT-29 proliferation and wound healing

**Figure 1:**
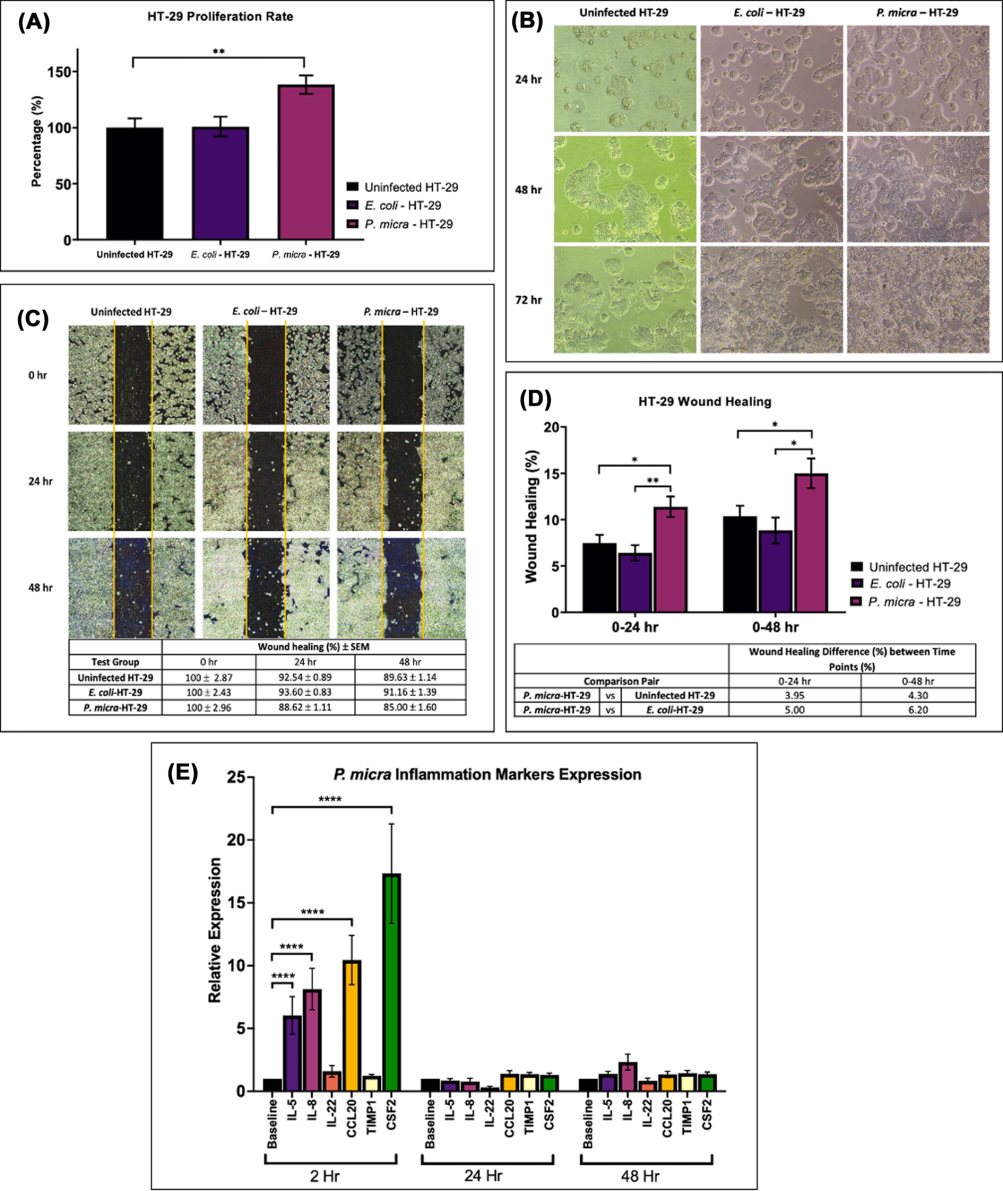
Effects of *P. micra* infection on cell proliferation, wound healing, and inflammation of the HT-29 cell line (**A**) Cell proliferation rate of HT-29 (uninfected HT-29), co-culture of *E. coli* DH5α and HT-29 (*E. coli*-HT-29), and co-culture of *P. micra* and HT-29 (*P. micra*-HT-29). Difference between groups were analyzed by Mann–Whitney test; **P≤ 0.01; (**B**) Observation of cell proliferation activity on uninfected HT-29, *E. coli*-HT-29, and *P. micra*-HT-29 at 24, 48, and 72 hr post-infection; (**C**) Wound healing and (**D**) wound area differences at 0–24 hr and 0–48 hr of uninfected HT-29 and HT-29 infected with *P. micra* and *E. coli*. Difference between groups were analyzed by Mann–Whitney test; *P≤ 0.05, **P≤0.01. (**E**) Relative expression of inflammatory markers (*IL-5*, *IL-8*, *IL-22*, *CCL20*, *TIMP1*, *CSF2*) upon *P. micra* infection on HT-29 at 2, 24, and 48 hr post-infection. Difference between groups were analyzed by two-way multiple comparison ANOVA test; ****P≤0.0001.

Co-culture of *P. micra* with HT-29 increased the rate of HT-29 proliferation by 38.45% (*P*=0.008) 72 hr post-infection (Figure 1A). Highly densed colonies were also observed on cells infected with *P. micra*. On the other hand, *E. coli* DH5α co-culture did not affect HT-29 cell proliferation (*P*=0.691) nor its colony formation. *P. micra* infection was found to increase HT-29 proliferation rate and promote morphological changes compared with experimental controls (Figure 1B).

The wound healing activity of infected HT-29 was determined at 0, 24, and 48 hr post-infection at a unified location (Figure 1C). Cells infected with *P. micra* showed highest wound healing percentage at 24 hr (10.55%) and 48 hr (12.7%) post-infection. At 24 hr, wound healing activity in *P. micra*-HT-29 was substantially higher compared to uninfected HT-29 (3.95%, *P*=0.02) and *E. coli*-HT-29 (5.00%, *P*=0.003). The same trend was observed at 48 hr comparison between *P. micra*-HT-29 against uninfected HT-29 (4.30%, *P*=0.03) and *E. coli*-HT-29 (6.20%, *P*=0.01) (Figure 1D).

### Induction of inflammatory response upon *P. micra* infection

Quantification of the expression of six inflammatory markers (*IL-5*, *IL-8*, *IL-22*, *CCL20*, *TIMP1*, *CSF2*) was carried out at 2, 24, and 48 hr post-infection. *P. micra* infection was found to up-regulate the expression of *IL-5* (*P*=0.0001), *IL-8* (*P*=0.0001), *CCL20* (*P*=0.0001), and *CSF2* (*P*=0.0001) at 2 hr post-infection (Figure 1E). Nevertheless, the increment in expression was not observed at 24 and 48 h post-infection. *IL-22* and *TIMP1* expression was found to be similar at all time-points point infection.

### Proteomics profiling of HT-29 infected with *P. micra*

Differential proteomics profiling analysis between uninfected HT-29 & *P. micra*-HT-29 revealed a total of 59,959 protein spectra. Eliminating the low-scoring spectra, 34,794 unique spectra were matched with 1,777 proteins based on the SwissProt human database. A FDR at 1% reduced the amount of unique proteins to 1,389. From these proteins, a total of 157 up-regulated proteins and 214 down-regulated proteins were identified (*P*<0.05).

### Gene Ontology (GO) enrichment analysis in *P. micra*-associated CRC tumorigenesis

**Figure 2:**
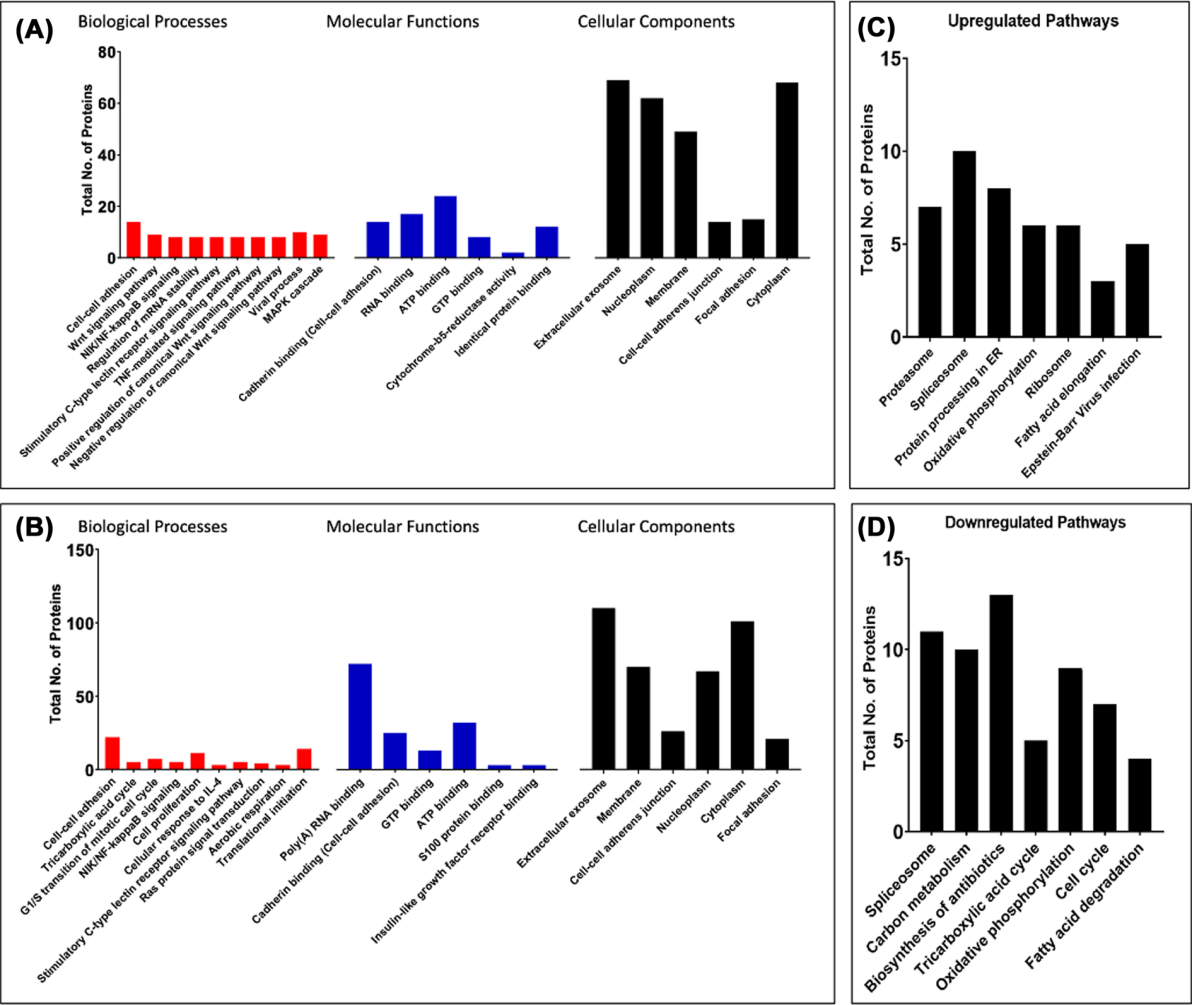
Gene ontology (GO) enrichment analysis of *P. micra* infection in HT-29 (**A**) GO enrichment analysis of up-regulated proteins in *P. micra*-HT-29 compared with uninfected HT-29 and (**B**) GO enrichment analysis of down-regulated proteins in *P. micra*-HT-29 compared with uninfected HT-29 based on biological processes, molecular functions, and cellular components. Enrichment analysis was made using DAVID Bioinformatics Resources 6.8 and PANTHER16.0; (**C**) Enriched up-regulated pathways and (**D**) enriched down-regulated pathways associated with *P. micra* infection in CRC tumorigenesis. Pathways enrichment was analyzed using DAVID Bioinformatics Resources 6.8 and PANTHER16.0.

GO enrichment analysis of up- (Figure 2A) and down-regulated (Figure 2B) proteins expressed during *P. micra*-associated HT-29 cell proliferation revealed differential protein expression of cellular components, correlating cellular processes changes during cell proliferation and wound healing.

### *P. micra* in CRC tumorigenesis

Up- and down-regulated differentially expressed proteins in *P. micra*-HT29 compared with uninfected HT-29 were analysed separately to reveal *P. micra*-associated CRC tumorigenesis pathways. Figure 2C shows seven differentially expressed pathways with the highest log2 fold change in *P. micra*-HT-29 compared to uninfected HT-29. On the other hand, pathways that were found to be down-regulated by *P. micra* infection are illustrated in Figure 2D.

**Figure 3:**
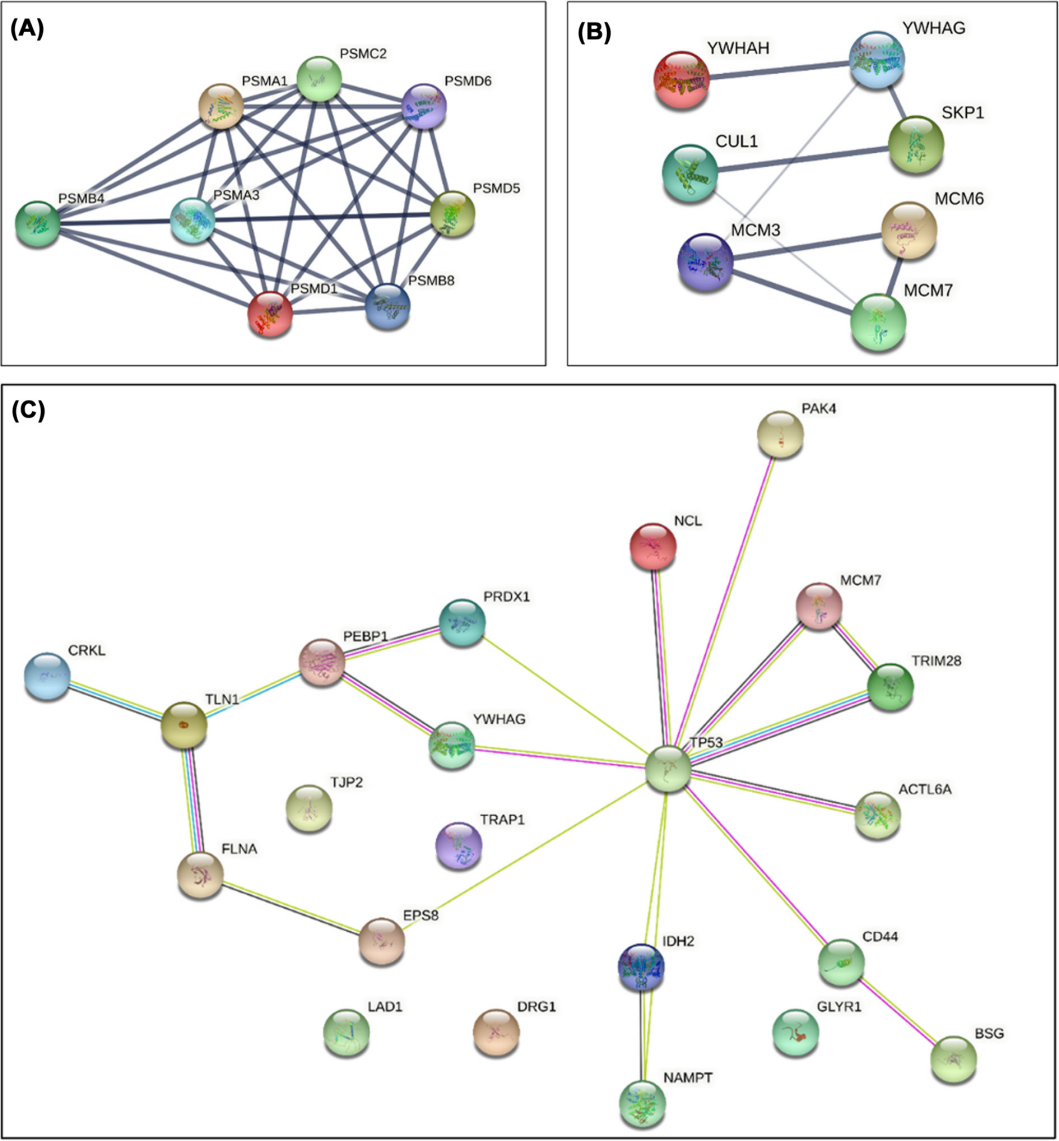
Protein-protein network interaction of *P. micra* infection in HT-29 (**A**) Protein network interaction of PSMB4 with neighbouring proteasomal subunits that were differentially expressed in *P. micra*-HT-29 based on STRING. (**B**) Protein network interaction of CUL1, YWHAH, and MCM7 with proteins associated with cell cycle activity in HT-29 infected with *P. micra* generated using STRING; (**C**) Network of EMT-related proteins in CRC tumorigenesis expressed by HT-29 upon *P. micra* infection generated from STRING.

Top 10 proteins with the highest log2 fold change from these seven pathways were further analyzed (Table 1). Proteasome subunit beta type-4, PSMB4, was identified to be putatively crucial in *P. micra*-associated CRC tumorigenesis and commonly overexpressed in other human cancer [23]. PSMB4 protein–protein interaction figure with neighbouring proteasomal subunits (PSMA1, PSMA3, PSMC2, PSMD1, PSMD6, PSMB8, and PSMD5) was built (Figure 3A, Table 2).

**Table 1:** Primer sequences for inflammatory marker and housekeeping gene expression determination

Marker	Primer	Nucleotide Sequences (5′ to 3′)	Length (base pair)
Inflammatory	*CSF2* Forward	5′-AATGTTTGACCTCCAGGAGCC-3′	21
	*CSF2* Reverse	5′-TCTGGGTTGCACAGGAAGTTT-3′	21
Inflammatory	*IL-5* Forward	5′-AACTGTGCAAGGGGGTACTG-3′	20
	*IL-5* Reverse	5′-AGGCCTGACTCTTTCTTGGC-3′	20
Inflammatory	*IL-8* Forward	5′-TGCTTCCCCTTAGCATTTTGT-3′	21
	*IL-8* Reverse	5′-CCAGCTATGCTAAAGCGCAC-3′	20
Inflammatory	*IL-22* Forward	5′-CCTTCCCCAGTCACCAGTTG-3′	20
	*IL-22* Reverse	5′-TGCGGTTGGTGATATAGGGC-3′	20
Inflammatory	*CCL20* Forward	5′-CAAGAGTTTGCTCCTGGCTG-3′	20
	*CCL20* Reverse	5′-GCTTGCTGCTTCTGATTCGC-3′	20
Inflammatory	*TIMP1* Forward	5′-TTCTGGCATCCTGTTGTTGCT-3′	21
	*TIMP1* Reverse	5′-CCTGATGACGAGGTCGGAATT-3′	21
Housekeeping	*HPRT* Forward	5′-TGTTGGTTCCATTTTCCTTGTTTG-3′	24
	*HPRT* Reverse	5′-GGTAGCCAAGTGGACCTCAG-3′	20
Housekeeping	*β-tubulin* Forward	5′- TTCAAGGGAGGTGTCAGCAGTA-3′	22
	*β-tubulin* Reverse	5′- GTGAGGGAGGTAGAGTTGGAA-3′	21

**Table 2:** Top 10 up-regulated proteins from pathways that were enriched by *P. micra* infection in HT-29

Pathway	Protein (Gene ID)	*P*-value	Log2 fold change
Oxidative phosphorylation	ATP5F1A	0.0008	1.67
Protein processing in ER	DNAJA1	0.03	1.64
Spliceosome	U2AF2	0.0008	1.57
Ribosome	MRPL10	0.02	1.50
Fatty acid elongation	TECR	0.01	1.49
Proteasome	PSMB4	0.0007	1.48
Ribosome	RPS28	0.002	1.41
Protein processing in ER	SEC24C	0.02	1.38
Spliceosome	LSM5	0.02	1.31
Protein processing in ER	CAPN1	0.009	1.31

**Table 3:** Top 10 down-regulated proteins from pathways that were enriched by *P. micra* infection

Pathway	Protein (Gene ID)	*P*-value	Log2 fold change
Oxidative phosphorylation	MT-CO2	0.01	--1.55
Fatty acid degradation	ACOX1	0.005	−1.44
Oxidative phosphorylation	ATP5PB	0.02	−1.42
Oxidative phosphorylation	NDUFS3	0.003	--1.42
Cell cycle	YWHAH	0.0007	−1.40
Cell cycle	CUL1	0.03	--1.39
Spliceosome	SNRPD1	0.01	−1.32
Oxidative phosphorylation	PPA2	0.002	--1.31
Spliceosome	RBM17	0.001	−1.30
Cell cycle	MCM3	0.02	--1.29

Among the top 10 down-regulated proteins in *P. micra*-HT-29 were cullin 1 (CUL1), tyrosine 3-monooxygenase/tryptophan 5-monooxygenase activation protein eta (YWHAH), and minichromosome maintenance complex component 3 (MCM3). Protein network interaction between these three proteins and other significantly down-regulated proteins (YWHAG, SKP1, MCM6, and MCM7) are shown in Figure 3B.

Intriguingly, a few apoptosis related proteins were also found to be differentially expressed by the infected cells. These include the up-regulation of heat shock protein family (HSAP9, HSP90B) and down-regulation of apoptosis-inducing factor 1 (AIFM1).

### Overexpression of epithelial–mesenchymal transition (EMT) proteins induced by *P. micra* in HT-29

Some of the differentially expressed proteins (*n*=47) in *P. micra* infected HT-29 were associated with EMT. Nine up-regulated (CD44, DRG1, BSG, NCL, CRKL, TP53, PAK4, NAMPT, ACTL6A) and thirteen down-regulated proteins (PRDX1, PEBP1, FLNA, EPS8, YWHAG, TRIM28, TLN1, IDH2, TRAP1, GLYR1, MCM7, LAD1, TJP2) were associated with CRC tumorigenesis (*P*<0.05). Protein–protein interaction network of these proteins elucidated TP53 regulation with significant interaction of NCL, PAK4, MCM7, TRIM28, YWHAG, ACTL6A, CD44, IDH2, EPS8, and PRDX1 during *P. micra* infection (Figure 3C).

## Discussion

Gut mucosal and faecal sequencing association studies have identified a variety of gut bacteria as being over-represented in CRC patients [12–14]. Nevertheless, mechanistic studies demonstrating tumorigenesis effects of these bacteria, including *P. micra*, a gut microbiota associated with CRC tumours of the consensus molecular subtype 1 (CMS1) [24], remains few. *P. micra* was previously known as *Streptococcus micros* (1933), *Peptostreptococcus micros* (1957), and *Micromonas micros* (1999) [25,26] and is the sole species in the *Parvimonas* genus. It is a Gram-positive cocci, an obligate anaerobe that resides dominantly in the oral cavity as a commensal pathogen. Even so, the isolation of *P. micra* is not limited to the oral cavity [27], but also in the laryngeal pharynx, gastrointestinal tract, pus abscess, spondylodiscitis, and blood samples [28–30].

In the present study, *P. micra* infection was accompanied by a significant increase in the proliferation of HT-29 for three consecutive days, compared with our control experiments comprising *E. coli*-HT-29 and uninfected HT-29 that showed no significant changes. In addition, co-culture of *P. micra* with HT-29 demonstrated significantly increased wound healing properties even at 48 hr observation. Similar observations were reported by Zhao et al. suggesting that the bacteria may trigger proliferative signalling in cells and lead to uncontrollable cell division [17].

Upon co-culturing *P. micra* and HT-29, the expression of *IL-5*, *IL-8*, *CCL20*, and *CSF2* became elevated; indicating the possible activation of PI3K-AKT, NF- κB and MAPK pathway in *P. micra*-associated CRC [31–34]. Indeed, most CRC-associated pathobionts have been shown to modulate inflammation. Enterotoxigenic *B. fragilis* was found to induce colonic tumours via the T-helper type 17 inflammatory response [8] and infection of *F. nucleatum* caused upregulation in cellular cytokines levels via NF-κB activation and β-catenin phosphorylation [35]. In the *in vivo* study by Yu et al. [18], *P. micra* infection of germ-free mice was found to increase the expression level of pro-inflammatory cytokines including TNF-α, IL-17A, IL-6, and CXCR1, indicating the importance of inflammation in pathobiont-associated CRC. Even though the increases were only observed at the 2 hr time point in our study, this infection-associated inflammation may sustain *in vivo*, especially in the colon of CRC patients. This occurs in addition to cell proliferation and migration, contributing towards tumorigenesis.

To further disentangle the roles of *P. micra* in CRC, differential protein expression between *P. micra*-HT-29 and uninfected HT-29 was determined. Proteins of cellular components in HT-29 were found to be enriched by *P. micra* infection and this corresponds to the proliferative changes observed in our functional assays. In addition, expression of proteins involved in NIK/NF-κB, Wnt signalling, and TNF-mediated signalling pathways [36,37] were also elevated in *P. micra* infected HT-29, indicating the carcinogenic role of the bacteria in tumorigenesis. Of note, up-regulation of PSMB4 was also found in *P. micra*-HT-29, which hypothetically occurred alongside the increased expression of neighbouring proteasomal subunits of PSMA1, PSMA3, PSMB8, PSMC2, PSMD1, PSMD5 and PSMD6. These protein components are crucial to the ubiquitin–proteasome pathway (UPP) activation in cancer [38,39], where they interfere degradation of β-catenin via Wnt signalling, resulting in continuous proliferation and metastasis in CRC [40,41]. PSMB4 was also found to be associated with formation of blood vessels (angiogenesis) and metastasis [42,43]. Upregulation of PSMB4 and mutated tumour suppressor p53 in the infected cells might function to activate inflammatory responses via NIK/NF- κB pathways [23,44]. On the other hand, down-regulation of CUL1, YWHAH, and MCM3 was observed; these proteins play a role in disturbing cell cycle activity [45–47]. Furthermore, significant differentially expressed EMT proteins were found to be induced by *P. micra* infection, mediating the cells towards increased proliferation, invasion and survival in CRC [48–53]. In addition, regulation of proteins such as HSAP9, HSP90B, and AIFM1 was altered, restricting apoptosis and promoting proliferation of infected cells [54–56].

**Figure 4:**
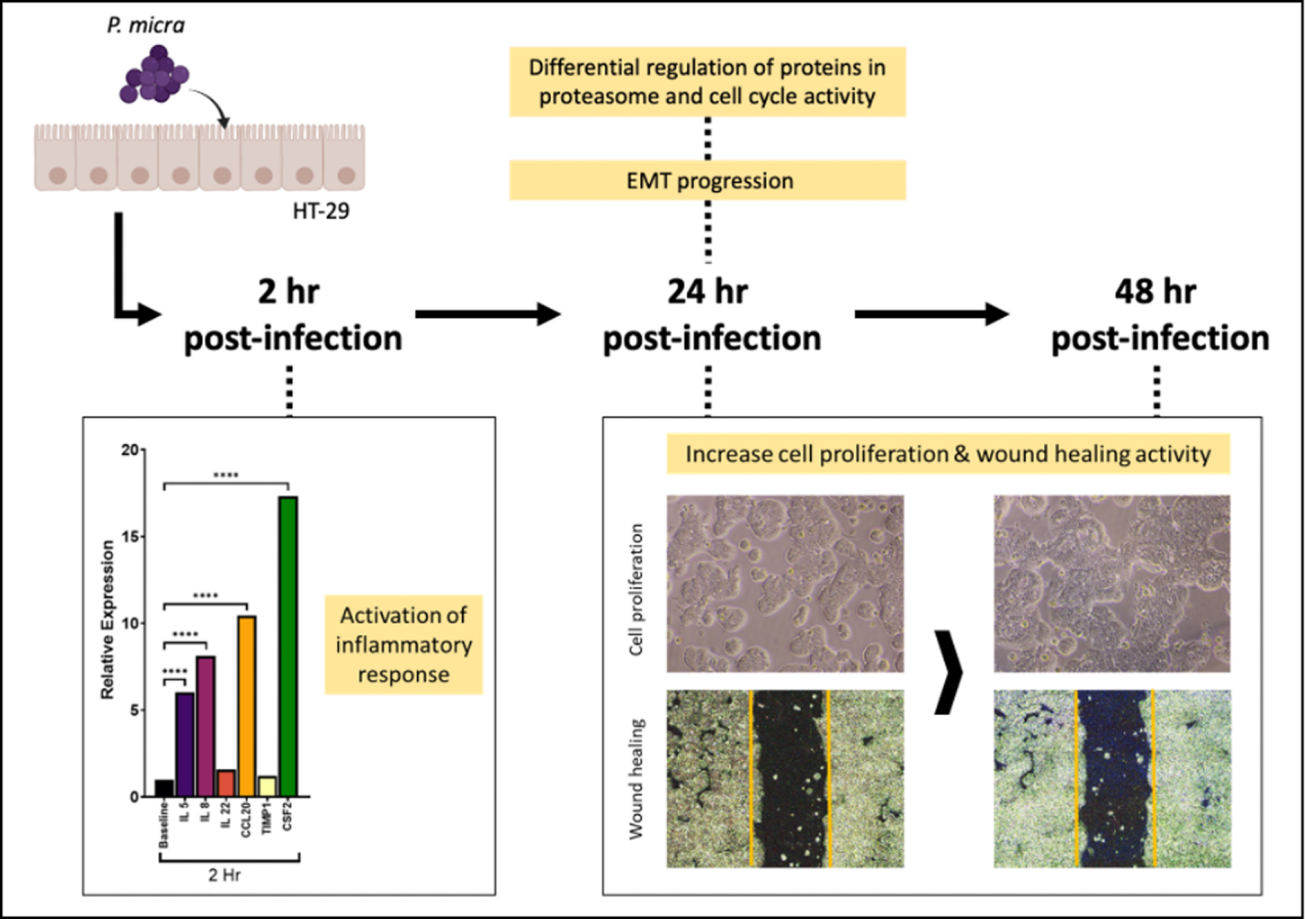
Summary of events in *P. micra*-associated tumorigenesis *P. micra*-associated carcinogenesis in HT-29 at different post-infection time points. Cell inflammation was detected at 2 hr, followed by cell proliferation and increased wound healing at 24 and 48 hr post-infection.

Taking it all together, we found that *P. micra* infection in HT-29 for 2 hr in an *in vitro* system lead to increased inflammatory response in the cells. This occurs in concert with cell perturbation due to proteasome and cell cycle activity, mediating the cells towards EMT at 24 hr after infection. Morphologically, increased cell proliferation and wound healing were observed compared to HT-29 cells without infection, signifying the contribution of *P. micra* towards increased tumorigenesis in HT-29 (Figure 4). Nevertheless, as these observations were found in an *in vitro* model utilizing a CRC cell line (whereas an *in vivo* model will allow investigation into the dynamic effects of chronic *P. micra* infection on the colon cells), future studies investigating similar parameters of the bacterial infection in an animal model will be useful to confirm the definite role of *P. micra* in CRC.

## Future direction

As this study has been conducted on the HT-29 (a CRC cell line) and with *P. micra* as the only tested pathobiont, future areas of investigation include to confirm the tumorigenic potential of the bacteria in a non-CRC colon cell line, both in a sole-pathobiont and also dysbiotic bacterial community study to elucidate the complexity and interaction of these bacterial communities in CRC. It will also be interesting to investigate the ability of probiotics in protecting cells infected with *P. micra* from tumorigenesis. As *P. micra* demonstrated tumorigenic properties in this study and has been identified to be over-represented in gut mucosal tissues and stool samples of CRC patients, the bacteria could be investigated as potential biomarker for CRC.

## Conclusions

In conclusion, this study demonstrated increased tumorigenesis properties caused by *P. micra* infection in a HT-29 model and provided additional evidence of *P. micra* as a pathobiont in the colon. Future studies of the bacteria's interplay with other colon pathobionts will be important to understand its role in CRC.

## Data Availability

Data are available on request. All data relevant to the study are included in the article or uploaded at http://dx.doi.org/10.13140/RG.2.2.24406.32320.

## Abbreviations

ACOX1: acyl-CoA oxidase 1
ACTL6A: actin like 6A
AIFM1: apoptosis-inducing factor 1
ATP5F1A: ATP sythase F1 subunit alpha
ATP5PB: ATP synthase peripheral stalk-membrane subunit B
BSG: basigin
CAPN1: calpain 1
CCL20: chemokine (C-C) motif ligand 20
CMS1: consensus molecular subtype
CRC: colorectal cancer
CRKL: CRK like proto-oncogene, adaptor protein
CSF2: colony stimulating factor 2
CUL1: cullin 1
CXCR1: C-X-C motif chemokine receptor 1
DNAJA1: DnaJ heat shock protein family (Hsp40) member A1
DRG1: developmentally regulated GTP binding protein 1
EMT: epithelial–mesenchymal transition
EPS8: epidermal growth factor receptor pathway substrate 8
FLNA: filamin A
GLYR1: glyoxylate reductase 1 homolog
GO: gene ontology
HPRT: hypoxanthine phosphoribosyltransferase 1
HSAP9: heat shock protein family A (Hsp70) member 9
HSP90B: heat shock protein 90 alpha family class B member 1
IDH2: isocitrate dehydrogenase (NADP(+))2)
IL-5: interleukin 5
IL-6: interleukin 6
IL-8: interleukin 8
IL-17A: interleukin 17A
IL-22: interleukin 22
iTRAQ: isobaric tag for relative and absolute quantitation
LAD1: ladilin 1
LSM5: LSM5 homolog, U6 small nuclear RNA and mRNA degradation associated
MCM3: minichromosome maintenance complex component 3
MCM6: minichromosome maintenance complex component 6
MCM7: minichromosome maintenance complex component 7
MOI: multiplicity of infection
MRPL10: mitochondrial ribosomal protein L10
MT-CO2: mitochondrially encoded cytochrome C oxidase II
NAMPT: nicotinamide phosphoribosyltransferase
NCL: nucleolin
NDUFS3: NADH:ubiquinone oxidoreductase core subunit S3
PAK4: P21 (RAC1) atctivated kinase 4
PEBP1: phosphatidylethanolamine binding protein 1
PPA2: inorganic pyrophosphatase 2
PRDX1: peroxiredoxin 1
PSMA1: proteasome 20S subunit alpha 1
PSMA3: proteasome 20S subunit alpha 3
PSMB4: proteasome subunit beta 4
PSMB8: proteasome 20S subunit beta 8
PSMC2: proteasome 26S subunit, ATPase 2
PSMD1: proteasome 26S subunit, non-ATPase 1
PSMD5: proteasome 26S subunit, non-ATPase 5
PSMD6: proteasome 26S subunit, non-ATPase 6
RBM17: RNA binding motif protein 17
ROS: reactive oxygen species
RPS28: 40S ribosomal protein S28
SEC24C: SEC24 homolog C, COPII coat complex component
SNRPD1: small nuclear ribonucleoprotein D1 polypeptide
SKP1: S-phase kinase-associated protein 1
TECR: trans-2,3-enoyl-CoA reductase
TIMP1: TIMP metallopeptidase inhibitor 1
TJP2: tight junction protein 2
TLN1: talin 1
TP53: tumor protein P53
TRAP1: TNF receptor associated protein 1
TRIM28: tripartite motif containing 28
UPP: ubiquitin–proteasome pathway
U2AF2: U2 small nuclear RNA auxiliary factor 2
YWHAG: tyrosine 3-monooxygenase/tryptophan 5-monooxygenase activation protein gamma
YWHAH: tyrosine 3-monooxygenase/tryptophan 5-monooxygenase activation protein eta
